# Regulation of Root Angle and Gravitropism

**DOI:** 10.1534/g3.118.200540

**Published:** 2018-10-15

**Authors:** Ted W. Toal, Mily Ron, Donald Gibson, Kaisa Kajala, Bessie Splitt, Logan S. Johnson, Nathan D. Miller, Radka Slovak, Allison Gaudinier, Rohan Patel, Miguel de Lucas, Nicholas J. Provart, Edgar P. Spalding, Wolfgang Busch, Daniel J. Kliebenstein, Siobhan M. Brady

**Affiliations:** * Department of Plant Biology and Genome Center; †† Deparment of Plant Sciences, UC Davis, California; † Department of Botany, University of Wisconsin, 430 Lincoln Drive, Madison, Wisconsin; ‡ Gregor Mendel Institute (GMI), Austrian Academy of Sciences, Vienna Biocenter (VBC), Dr. Bohr-Gasse 3, 1030 Vienna, Austria; § Department of Cell and Systems Biology, Centre for the Analysis of Genome Evolution and Function, University of Toronto, 25 Willcocks St., Toronto, Ontario, Canada; ** Salk Institute for Biological Studies, Plant Molecular and Cellular Biology Laboratory, 10010 N Torrey Pines Rd, La Jolla, Califronia

**Keywords:** gravitropism, root, tomato

## Abstract

Regulation of plant root angle is critical for obtaining nutrients and water and is an important trait for plant breeding. A plant’s final, long-term root angle is the net result of a complex series of decisions made by a root tip in response to changes in nutrient availability, impediments, the gravity vector and other stimuli. When a root tip is displaced from the gravity vector, the short-term process of gravitropism results in rapid reorientation of the root toward the vertical. Here, we explore both short- and long-term regulation of root growth angle, using natural variation in tomato to identify shared and separate genetic features of the two responses. Mapping of expression quantitative trait loci mapping and leveraging natural variation between and within species including Arabidopsis suggest a role for *PURPLE ACID PHOSPHATASE* 27 and *CELL DIVISION CYCLE 73* in determining root angle.

Directing growth toward optimal conditions is critical to plant survival. Roots must grow toward water, nutrients, and physical support, while simultaneously avoiding growth toward hostile, inadequate, and non-supportive environments. To that end, the plant must integrate numerous environmental and internal signals to direct root tip and growth angle in a way that will place the root in an environment capable of nurturing sustainable long-term growth. A root’s final angle is the net result of a series of responses to these stimuli. As such, regulation of root angle is an important trait for plant breeding and has been correlated with increased yield (de Dorlodot *et al.* 2007; Lynch 1995).

Among the many environmental conditions to which a plant must sense, integrate and respond, gravity is a central input. Gravitropism, a directed short-term growth response to gravity, has been extensively genetically and mechanistically studied in *Arabidopsis thaliana* (Müller *et al.* 1998; Chen *et al.* 1998; Marchant *et al.* 1999; Mullen *et al.* 1998; Luschnig *et al.* 1998; Fukaki *et al.* 2002; Kerwin *et al.* 2015). In a short-term response to gravity, dense starch-containing amyloplasts in the columella cells at the root tip sediment downward via dynamic interactions with vacuolar membranes and the cytoskeleton (Morita 2010). This triggers a signaling cascade (Baldwin *et al.* 2013; Toyota and Gilroy 2013; Kimbrough *et al.* 2004) that alters the localization of auxin PIN efflux transporters (Sack 1997; Friml *et al.* 2002). The outward-flowing auxin transport via AUX, PIN, and ABCB transporters at the root tip becomes asymmetric, leading to an auxin concentration difference across the root. A subsequent auxin signaling cascade leads to altered epidermal cell elongation, with the side of the epidermis having the higher auxin level exhibiting a lower elongation rate, resulting in the root tip bending downward (Band *et al.* 2012; Spalding 2013; Swarup *et al.* 2005). The shifted auxin accumulation also interacts with a complex signaling network including signaling from cytokinin, ethylene, brassinosteroid, jasmonate, strigolactone, and glucose, enabling multiple environmental inputs to influence gravitropism (Kushwah *et al.* 2011; Zhou *et al.* 2011; Kim *et al.* 2007; Koltai 2015; Gutjahr *et al.* 2005; Zheng *et al.* 2011; Sang *et al.* 2014).

In *Arabidopsis thaliana*, another type of root growth that involves long-term interpretation of the gravity vector is wavy growth in a particular direction (usually right-handed) that occurs when roots are grown on a vertical, hard agar surface. Arabidopsis roots grown on hard agar plates inclined to the vertical produce a pattern of waves whose slanting to the right side is enhanced relative to the directionality on a vertical agar plate. This phenomenon is referred to as slanting or skewing. Okada and Shimura (Okada and Shimura 1990) initially interpreted this as the consequence of an interaction between thigmotropism (bending in response to touch) and positive gravitropism. Right-handed slanting was first reported by Simmons *et al.* (Simmons *et al.* 1995). and subsequently, slanting mutants were reported by Rutherford and Masson (Rutherford and Masson 1996). Circumnutation, or a revolving nutation, is a type of bending movement that is created by unequal growth rates on different sides of the organ (Migliaccio *et al.* 2013). Migliaccio and Piconese interpreted slanting on tilted agar plates as the result of an interaction between right-handed circumnutation (defined with respect to the right-handedness of the root helix), positive gravitropism and negative thigmotropism (Migliaccio and Piconese 2001). Recent employment of computer automation is providing accurate high-throughput measurement of the short-term gravity response, allowing an increasing characterization of novel gravitropism components (Miller *et al.* 2007; Brooks *et al.* 2010) in *Arabidopsis thaliana* and revealing the presence of additional unidentified players in a root’s response to gravity.

Root growth angle is regulated not only by a response to gravity. Root angle can also be regulated in response to nutrient availability and temperature (Niu *et al.* 2015; Ritchie *et al.* 1997; Massa and Gilroy 2003; Bonser *et al.* 1996; Nakamoto 1995). Regulation of root angle has been previously linked to drought avoidance in rice (Uga *et al.* 2011). It is conceivable that plant species have employed distinct genetic mechanisms to regulate a root’s response to gravity differently from regulation of root growth angle in response to specific environments. This could include different short-term and long-term signaling processes to optimize root growth angles for nutrient and water acquisition. Here, we explore the genetic basis of root angle relative to a root’s response to gravity in the context of natural variation.

Tomato (*S. lycopersicum*) is an important food crop whose root development differs in many aspects from the model species, *A. thaliana*, including root angle in the cultivar M82 (Ron *et al.* 2013). *S. pennellii* is an inter-crossable wild relative adapted to growth in dry, saline and rocky desert conditions, diverging from tomato some seven million years ago (Nesbitt and Tanksley 2002; Peralta *et al.* 2005). Root angle was previously demonstrated to differ between the accessions of these two species (long-term regulation) (Ron *et al.* 2013). Furthermore, 24 hr after rotating a plate 90 degrees, the resulting angle was the same as that prior to the rotation (short-term regulation) (Ron *et al.* 2013). While the root’s net angle requires the transduction of the gravity vector into growth control, it is unclear if this angle is established differentially in a dynamic fashion in the short-term and maintained over the long-term using the same genetic mechanisms. To identify genetic loci that may independently control root angle over these time scales, we have exploited natural variation in these traits within tomato.

We thus further explore the long-term regulation of root net angle between M82 and *S. pennellii* using a more sensitive and quantitative descriptor after several days of plant growth (Figure 1A). To determine if the genetic basis of the long-term regulation of root growth angle is the same or different from the short-term response to a change in gravity stimulus, we utilize “computer vision” to determine the dynamic genetic basis of root gravitropism over the short-term. This automated high-throughput approach allows the calculation of the rate of change of the root tip angle at discrete time points over two hours of root tip growth following a 90° rotation of a vertically-oriented plate (Figure 1D).

**Figure 1:**
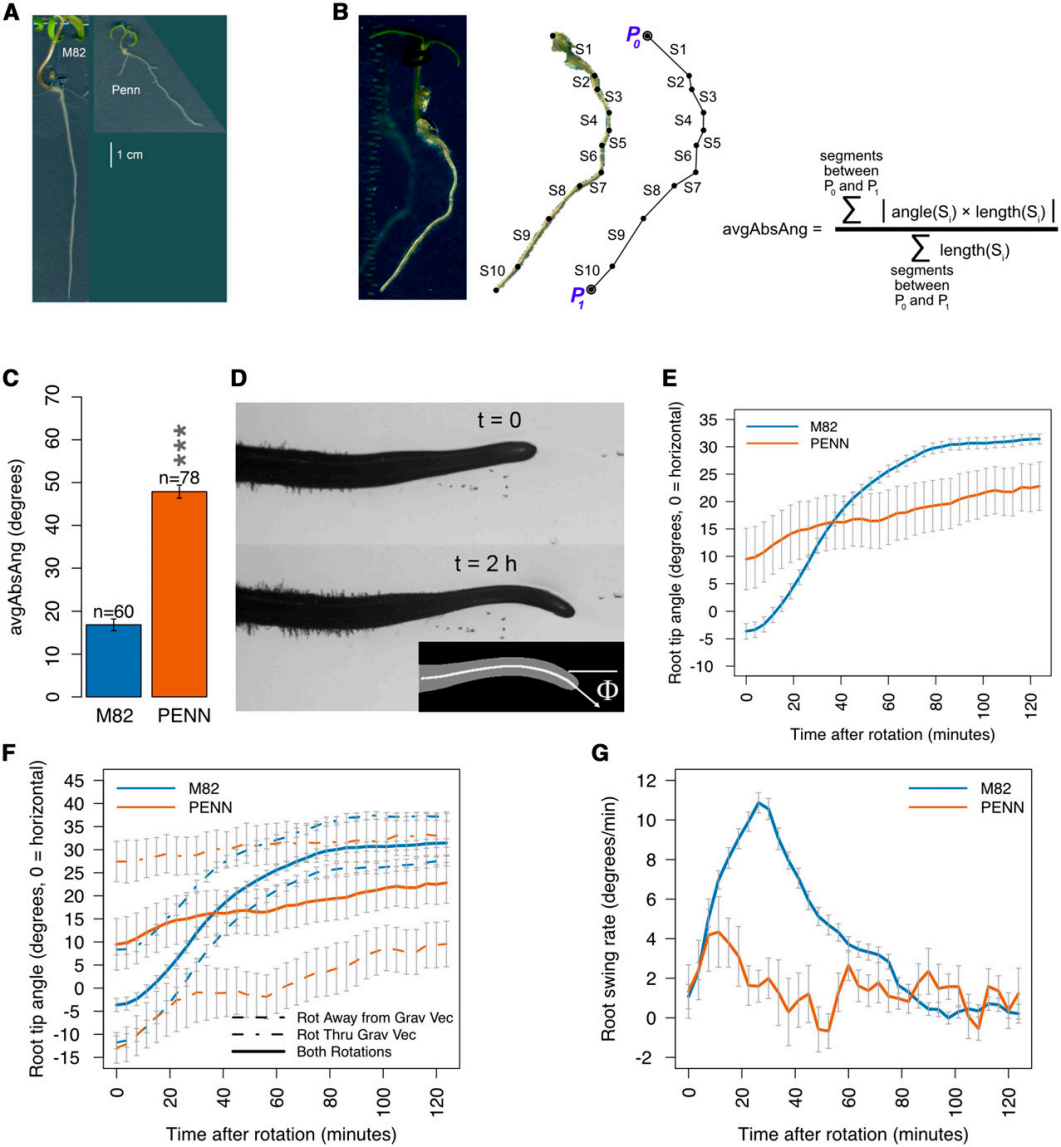
Average absolute angle and gravitropism in M82 and *S. pennellii*. (A) Roots of M82 (left) and *S. pennellii* (right). Scale bar = 1 cm. (B) Quantification of average absolute angle. (C) *S. pennellii* has a significantly greater average absolute angle relative to M82. ***= *P* < 0.001 (ANOVA). (D) Gravitropism was measured as the rate of tip angle change over two hours following a 90° rotation, with 0° as horizontal to the right and positive angle clockwise (as the tip bends downward). (E) Mean tip angle of M82 (n = 89) and *S. pennellii* (n = 18) as a function of time after rotation by 90 degrees. Error bars represent standard error of the mean. (F) Mean tip angle curve for all rotated (Rot) roots (solid) has the same response curve shape as, and is intermediate between, curves for roots rotated (rot) *through* or *away from* the gravity vector (dashed). M82: n = 36 *away*, n = 53 *through*; *S. pennellii*: n = 10 *away*, n = 8 *through*. Error bars represent standard error of the mean. (G) Mean rate of change of tip angle (swing rate), per time point in the two hours following 90 degree rotation of M82 (n = 89) and *S. pennellii* (n = 18). Error bars represent standard error of the mean.

Using quantitative genetic and transcriptomic approaches, we mapped quantitative trait loci and expression quantitative trait loci underlying differences in root angle in the short-term and the long-term between the accessions from each of these Solanum species. Together, these data demonstrate that the short-term and long-term regulation of root angle is largely genetically distinct. Using this genetic and gene expression resource across 76 introgression lines containing the *S. pennellii* genome introgressed into the *S. lycopersicum* genome, as well as a Genome Wide Association Study in *Arabidopsis thaliana*, we identify two novel players in regulation of root angle, *PURPLE ACID PHOSPHATASE* 27 *(PAP27)* and *CELL DIVISION CYCLE 73*.

## Methods

### Plant growth conditions – average absolute angle and RNAseq analysis

Seedlings of *S. lycopersicum* (cv. M82), *S. pennellii* (LA0716), and ILs were grown on Murashige and Skoog (MS) plates containing 4.3g L^-1^ MS, 0.5gL01 2-(N-morpholino) ethanesulfonic, 10g L^-1^ Suc, pH = 5.8, and 8g L^-1^ Agar. Seeds were placed in tissue-embedding cassettes and surface sterilized in 70% ethanol for 2 min, then in 3% hypochlorite for 20 min, followed by three washes with distilled water. Seven sterilized seeds were plated on each MS plate in a row 2.5 cm from the top of a square 12- x 12-cm plate and then sealed with 3M surgical tape. A minimum of four biological replicates of each of 79 genotypes were grown independently at four different times with 2 weeks between growth cycles for each of the four replicates. Genotypes included 73 of the 76 ILs of the IL population (1-1, 3-3, and 6-2-2 lacked seeds), the two parents *S. lycopersicum* (cv. M82) and *S. pennellii* (LA0716), and four sub-ILs: 1-1-4, 7-5-P5, 7-5-P75, and 7-5-1. Seven plate holders (A through G) each with slots to hold 12 pairs of plates (1A/1B through 12A/12B) were used, with plates paired so that their bottom sides faced one another. Each plate had 7 seeds oriented so the radicle would emerge from the seed in a downward direction. Each IL was plated on 2 plates for 14 seeds total (*i.e.*, per biological replicate), except M82 and *S. pennellii* were plated on 4 plates. For 79 genotypes this totals 79*2 + 4 = 162 plates, leaving 6 empty plate holder slots. Independently for each replicate, each IL was randomly assigned to a plate holder and plate position, using an Excel macro making use of the rand() function to randomly permute the list of ILs for the replicate. The 2 plates of each IL were placed into the same plate holder slot, back-to-back (positions A/B), and the 6 empty plate holder slots were filled with extra plates containing seeds. The 7 plate holders were placed on one shelf in the growth chamber, positioned and oriented so as to give each plate the same exposure to light and surrounding space. The experiments were carried out in a growth chamber with a 16:8 light:dark cycle at 22° and 50–75% humidity with a light intensity of 100μE. A harvest day was chosen for each plate by choosing the day that maximized the number of plate seeds that were 3 to 4 days post-germination. Immediately prior to harvesting root tissue for RNA-seq, plates were scanned with an Epson Perfection V700 photo flatbed scanner into 24-bit RGB TIF image files at 300 dpi. Plates were opened only once, at harvest time, to ensure the same growth conditions for all seedlings. Sampling occurred mid-afternoon. Between 7 and 14 root tip segments (1cm), 3 to 4 days post-germination, from each genotype replicate were cut and immediately placed into a labeled 2ml tube containing silica beads and immersed in liquid nitrogen. Samples were stored at −80° until library preparation.

### Image analysis, phenotype measurement, mixed effect linear model and ANOVA

Each replicate of each genotype had between 2 and 5 plates, all of which were scanned as described above. Images were analyzed with ImageJ (Rasband 1997-2014) supplemented with custom macros. Based on germination date and scan date, seedling age in days after germination was determined, and primary roots of seedlings that were 3 or 4 days post-germination were traced with the imageJ polyline tool, following the root centerline from the top of the root (the hypocotyl/root junction) to the tip along with metadata including genotype, plate ID, plating date, germination day of each seedling, plate harvest date, measurer, and two flags for each root: “collide” was true if the root contacted or crossed another root, and “along” was true if the root contacted another root and grew along it for any distance. Additional data were calculated from the metadata: root age (days from germination to harvest), “germination age” (days from plating to germination), “plating age” (days from plating to harvest), and biological replicate number The polylines in the data were analyzed to measure root average absolute angle (Figure 1B).

The derived angle data were used to fit the mixed-effect linear model trait ∼ genotype + collide + (1|germAge) + (1|plateDate), using the R lm() function in the stats package [68], the lmer() function in the lme4 package (Bates *et al.* 2015), and the lme() function in the nlme package [69]. The p.adjust() function was used to perform FDR adjustment of p-values using the “fdr” method [70]. Significance was defined as either p_FDR_ ≤ 0.01 or p_FDR_ ≤ 0.05 depending on the analysis (Table S1). All phenotype analysis code, macros, input data, augmented data, and result files are included in a supplemental dataset (S1_Dataset.zip).

Four ILs from the 2013 experiment (Ron *et al.* 2013), 6-1, 6-2-2, 8-2 and 9-1, along with M82 samples from that experiment, were measured for avgAbsAng as described above, and the angle data were used to fit the mixed-effect linear model trait ∼ genotype + (1|plateDate) using the lme() function, and p.adjust() was again used to perform FDR adjustment of p-values using the “fdr” method. Significance was defined as p_FDR_ ≤ 0.01 (Table S1).

### Gravitropic Rotation experiment

#### Growth and plating:

Tomato seeds placed in perforated plastic cassettes were surface-sterilized by soaking for 2 min in 70% ethanol, then for 15 min in a 50% household bleach solution. The seeds were then rinsed 6 times with sterile distilled water and immediately moved to sterile filter paper wetted with sterile distilled water contained in polystyrene Petri dishes. The dishes were placed horizontally in a continuously lit growth chamber for a minimum of 24 hr. Upon emergence of the radicle, seeds were placed on MS salts + 1% sucrose + 0.8% agar media. The agar plates were maintained vertically in a continuously lit growth chamber for a minimum of 24 hr, after which seedlings with roots between 10 and 20 mm were moved to a new plate of MS media in such a way as not to alter the initial angle of growth. These plates with seedlings were mounted in the imaging platform and allowed to recover for 1 hr.

#### Rotation, imaging, image processing, trait measurement:

Vertically-oriented plates containing seedlings were rotated +90° (counter-clockwise) about an axis perpendicular to the plate, so that a seedling growing at an angle of ±A° to vertical (positive angle counter-clockwise) attains an orientation of +90 ± A° to vertical, or **∓** A° to horizontal (positive angle clockwise, thus the sign flip). Plates were rotated in a direction that was random with respect to whether the pre-rotation angle of any given seedling was clockwise or counter-clockwise of straight down, hence the initial angle after rotation, when averaged across many seedlings, is close to zero.

Images were recorded every 3.75 min a total of 34 times over the course of two hours immediately at and following 90 degree rotation. Images were analyzed using a high-throughput automated image acquisition and analysis system for root tip angle and tip angle rate of change (Moore *et al.* 2013).

#### Linear model fitting and ANOVA:

Model fitting for the gravitropic rotation experiment was done using R ( Team 2014). The lm() function in the stats package fit the model “trait ∼ genotype” to each individual time point for both the tip angle and derivative traits, and the p.adjust() function was used to perform FDR adjustment of p-values using the Benjamini-Hochberg method. Time points with p_FDR_ ≤ 0.01 were considered significant. All rotation analysis code, input data, and result files are included in a supplemental dataset (S2_Dataset.zip).

### RNA-seq experiments

#### RNA-seq for germination day expression variation testing:

Due to the inability to synchronize germination in M82 seeds and seed limitation, we explored expression variation in 3-, 4- and 5-day-old seedlings. M82 seedlings were grown as described above, and harvest date for each plate was chosen to provide 7 to 10 seedlings on one plate for each of the three germination ages, and 7 to 10 root tips for each seedling age were collected as described above.

RNA-seq libraries were prepared from four replicates of M82 for each of the three root ages.

Libraries were created using a custom high-throughput method for Illumina RNA-seq with a poly-A enrichment step (Kumar *et al.* 2012). Libraries were pooled and sequenced in 50 bp single-end format at the UC Berkeley Genomics Sequencing Laboratory on two lanes of the HiSeq 2000 platform (Illumina Inc. San Diego, CA, USA).

Reads from the sequencing were demultiplexed, filtered for quality, and mapped to the Heinz cDNA genome version ITAG2.3, obtained from SolGenomics.net (www.solgenomics.net) (Fernandez-Pozo *et al.* 2015). The R package edgeR (Robinson *et al.* 2010) was used to normalize the mapped read counts using the TMM method (Robinson and Oshlack 2010), to estimate overdispersion and tagwise dispersion (Robinson and Smyth 2007), and then to call differentially expressed (D.E.) genes between each of the three pairs of conditions (3-4, 3-5, and 4-5 days post-germination). The topTags() “fdr” method (Benjamini and Hochberg 1995) was used to adjust for multiple testing (pFDR ≤ 0.01).

The number of D.E. genes between each pair was small, and the differences between each pair were small, with the largest difference deemed to be between the 3- and 5- day samples. Days 3 and 4 were chosen as germination days for subsequent RNA-seq sampling.

### IL RNA-seq library preparation and sequencing

The same root tissue harvested for measuring average absolute angle, described above, was used for the RNA-seq libraries, which were created using the Kumar *et al.* protocol with one modification: NEXTflex-96 adaptors were used. Library enrichment was done using 12 cycles. Each library was barcoded, using a unique bar code for each IL, but the same barcode for all replicates of an IL. The libraries of each replicate were pooled, and the four pools were purified with Ampure beads. The library pooling was tested using a Bioanalyzer.

Each replicate’s libraries were pooled and sequenced in 50 bp single-end format on a total of 5 lanes of a HiSeq 2000 platform (Illumina Inc. San Diego, CA, USA) at the UC Berkeley Genomics Sequencing Laboratory. Two replicates were lost during library creation, *pennellii* #3 and IL7-5 #3, and the remaining three replicates were deemed sufficient for the experiment.

### RNA-seq data filtering

Perl (Wall 1987-2012) scripts were written to check sequencing read files for expected bar codes, discarding questionable reads, masking low-quality bases, and demultiplexing the reads. RNA-seq reads containing polyN sequences of length ≥15 or containing significant fragments of either the forward or reverse adapter or their reverse complements were discarded. Bases with quality scores ≤ 20 were masked as N’s, and leading or trailing N’s were trimmed from the reads. Any two N’s within the read separated by fewer than 20 bp of non-N were trimmed and the read split into two reads at that point, with the longest piece retained. Trimmed reads with fewer than 10 non-N bases at the start or end, or with length < 30, were discarded. Libraries were demultiplexed by matching the read bar code to the library bar code or any code obtained from it by a single bp change including a change to N. FASTQC (Andrews 2011) was applied to the demultiplexed read files and its output examined for problems. Two test lanes were run for each pool for genotyping and double-checking library concentrations, before running the final three lanes, with all useable reads retained.

### Genotyping

We genotyped all libraries using the sequencing data. Matched references for *S. lycopersicum* cv. M82 and *S. pennellii* (Koenig *et al.* 2013) were concatenated into a single reference, and reads mapping with BWA (Li and Durbin 2009) to more than one position were discarded, leaving reads uniquely mapping to one or the other accessions of the two species. These reads were plotted and compared to the expected introgression positions of each IL.

### Chimeric references, read mapping, D.E. calling, and cis/trans classification

The approximate genomic positions of each IL’s introgression, from (Chitwood *et al.* 2013) for most ILs and via IL genotyping for a small subset of ILs (1-1-4, 7-5-P5, 7-5-P75, and 7-5-1), were used to combine the two matched references into a single *chimeric* reference containing the M82 sequence everywhere except in the introgression, where the *S. pennellii* sequence was used. Genes located at the introgression boundaries may have suspect expression counts, particularly in IL7-5-1, IL7-5-P75, and IL7-5-P5. Reads were mapped to the chimeric references using BWA (Li and Durbin 2009) with arguments “aln -t 4 -k 1 -l 25 -n 0.1 -e 15 -i 10”. Mapped reads were counted per file with samtools (Li *et al.* 2009), and R code was used to gather and combine all count files. The R package edgeR (Robinson *et al.* 2010) was used to normalize the mapped read counts using the TMM method (Robinson and Oshlack 2010) and normalized counts were summed over replicates for each genotype to provide mean expression data for later use. Overdispersion and tagwise dispersion were estimated with edgeR (Robinson and Smyth 2007), and differentially expressed (D.E.) genes were called between each genotype and M82 or PENN. The topTags() “fdr” method (Benjamini and Hochberg 1995) was used to adjust for multiple testing (pFDR ≤ 0.01). Each D.E. gene was categorized as *cis* or *trans* (Figure 3A) based on its known genomic position and the known positions of each introgression.

### Testing IL bins for D.E. genes consistent with a trait

A D.E. gene was defined as consistent with a trait if the gene is significantly differentially expressed in relation to M82 in at least two significant ILs of the gene’s bin, and the direction of the change in expression relative to M82 over all those significant ILs is consistent with the trait. To be consistent with the trait, the expression was either positively or negatively associated with the trait in all significant ILs for that trait – *i.e.*, an increase in expression (relative to M82) corresponds to an increase (positive) or decrease (negative) in the value (relative to M82). A minimum mean normalized expression count of 5 was required for a gene to be tested.

### Validation of Sl/SpPAP27 as an eQTL using qPCR

Real-time PCR primer pairs were designed using Primer-BLAST (Ye *et al.* 2012). Reference gene primers (Solyc01g014230): F, 5′ AGATTTGATGGACCCTGCTACCG-3′ and R, 5′- TCTTGACCGATTCCTGCTCTTCC-3′; PAP27 primers: F, 5′- CCCATTTACCAGAATCAATGTGT-3′ and R, 5′-TGGTGGTGAATCTATTCAAATGAG-3′. Amplification was performed with a MyIQ Real-Time PCR Detection System (BIO-RAD) using SyberGreen Dye (Biorad) and the following program: cycle 1, 10 min at 95°; cycle 2, 45 times 20 s at 95° followed by 30 s at 60° for Ref gene and 62° for PAP27; cycle 3, 15 s at 95°; and cycle 4, 70 times 30 s at 55°. The final volume of the PCR was 20 µl. The U-box domain-containing protein 4 (Solyc01g014230) was used as an internal control to normalize for variation in the amount of cDNA template. Each real-time PCR experiment contained three technical replicates. Three biological replicates (RNA from independently harvested tissues) were used. The relative gene expression levels were calculated using the 2^–∆∆t^ method (Livak and Schmittgen 2001).

### Measuring average absolute angle in Col-0 overexpression lines of PAP27

The *AtPAP27* gene *(AT5G50400)* was amplified from Col-0 cDNA using primers at the start and stop codons and cloned into D-TOPO and sequence validated. Primer sequences are found in Table S11. Resulting pENTR clones were recombined via LR into pK2GW7, which contains a 35S promoter. These constructs were transformed into *Agrobacterium tumefaciens* strain GV3101 and Col-0 plants were transformed via the floral dip method. T1 transformants were selected on MS media containing kanamycin, and reselection was repeated with T2 transformants. To confirm that the construct increased expression of *PAP27*, RT-qPCR was performed on the T3 transformants.

### Quantifying expression in PAP27 overexpression lines

Total RNA was extracted from P*AP27* overexpression lines and Col-0 controls approximately 7 to 10 days after germination. cDNA for each biological replicate was synthesized from four to six Arabidopsis seedlings using an adapted protocol in (Kumar *et al.* 2012). Amplification was performed on CFX96 Touch Real-Time PCR Detection System (Biorad) using SyberGreen Dye (Biorad) using the following program: cycle 1, 2 min at 95°, cycle 2, 40 times 5 sec 95° followed by 60° for 30 sec, cycle 3, 5 sec 95° followed by 60° for 5 sec. Primer sequences are found in Table S8. EF1a, was used as an internal control for variation in cDNA. Three to five biological replicates and four technical replicates per biological replicates were used. The relative gene expression levels were calculated using the 2^–∆∆t^ method (Livak and Schmittgen 2001).

### Determination of orthology for PAP27 homologs

BLAST+ (Camacho *et al.* 2009) was used with the protein sequence for Solyc07g008570 against the *S. lycopersicum* ITAG2.3 genome(Bombarely *et al.* 2011; Tomato Genome Consortium 2012) to identify five highly similar paralogs, and again against the *S. pennellii* V2.0 genome(Bolger *et al.* 2014) to identify seven highly similar homologs, and a third time against the *A. thaliana* TAIR10 genome(Lamesch *et al.* 2012; TAIR10 2013) to identify three highly similar homologs, the most similar being AT5G50400 (Table S9). Two more homologs per species were identified with BLAST+, having lower identity to the query sequence, for use as outgroup sequences. AT5G50400 was used to query EnsemblPlants (Kersey *et al.* 2014) for a phylogenetic gene tree (EBI 2015) precomputed using homology to the protein sequence of AT5G50400 (Vilella *et al.* 2009). The tree was used to identify two *A. thaliana* paralogs and six *S. lycopersicum* homologs, which were the same ones identified with BLAST+ (Figure S5). The protein sequences of all 16 homologs were multiply aligned with Muscle (Edgar 2004). A maximum likelihood phylogenetic tree was constructed with 200 bootstraps using MEGA (Tamura *et al.* 2011) from the aligned sequences. To confirm relationships shown in this tree, we used protein sequences from all these genes and the next two best BLAST hits from each of the three species to serve as outgroups, for a total of 22 protein sequences, to do a multiple alignment and generate a maximum likelihood tree (Figure S6). Based on these two trees, we identified *PAP27 (AT5G50400)* as the most similar Arabidopsis gene to *Solyc07g008570*. Also based on these trees, we assigned working symbolic names to the genes (Table S9).

### PAP27 homolog rearrangements

Close study of the alignment of the PAP27 homologs in M82 and *S. pennellii* showed that the Sopen07g004470 region containing exons 6-11 appears to have undergone a tandem duplication to the region containing exons 12-17 of the same gene and another tandem duplication to exon 6-11 region of Sopen07g004480. Solyc07g008550 has 11 exons, and exon region 5-11 maps well to Sopen07g004470 exon region 5-11. There is no sign of Sopen07g004480s exon 1-5 region in *S. lycopersicum*, but Sopen07g004470s intron 1-2 region maps well to Solyc07g008550 intron 1-2 region.

### Arabidopsis root angle genome wide association analysis

#### Plant material and growth conditions:

Seeds of 257 *Arabidopsis thaliana* accessions (Table S6) from the RegMap panel ***(******Horton et al. 2012******)*** were surface sterilized for one hour with chlorine gas generated from 130 mL of 10% sodium hypochlorite and 3.5 mL of 37% hydrochloric acid. For each accession, 24 seeds were plated over eight plates in a permutated block design to account for positional and within-plates effects. Seeds were placed on the surface of 50 mL 1x MS medium pH 5.7 with added 0.8% (w/v) agar (Duchefa Biochemie), 1% (w/v) sucrose. Seeds on plates were stratified 3 days at 4° in the dark, then plates were positioned vertically in tight racks. Racks with plates were then transferred for germination and growth to a chamber with constant 21° and a 16h-light/8h-dark cycle. Data for a subset of these lines (163 accessions) has been published in (Slovak *et al.* 2014).

#### Scanning, phenotyping, and direction index/root angle analysis:

Color images at 1200 dpi were acquired on day 4 using a cluster of Epson Perfection V600 flatbed scanners (Seiko Epson). The cluster of scanners was operated by the BRAT image acquisition tool to speed up the acquisition process and acquire 8 scans simultaneously (Slovak *et al.* 2014). TIFF images at 1200dpi 8-bit RGB were processed by BRAT software (Slovak *et al.* 2014), a FIJI plug-in (Schindelin *et al.* 2012). BRAT image acquisition tool and software executed unsupervised image segmentation and root detection, supervised quality control and subsequent automatic trait evaluation. The direction index trait is calculated as a sum of values assigned at each pixel of the main root from the hypocotyl/root junction toward the root tip (straight downward: 0, diagonal downward: 1, straight left or right: 2, diagonal upwards: 3, upwards: 4) (Slovak *et al.* 2014). The total sum is then divided by the number of pixels visited. Root angle is calculated as the angle between root vector (specified by the hypocotyl/root junction and the root tip projected on the root topological skeleton) and the vertical axis of root picture (assumed vector of gravity) [°].

#### Genome wide association mapping for average root direction in A. thaliana:

Median root direction index of 257 accessions (n = 7 to 24) quantified by BRAT (n = 7 to 24) was used for GWAS(Slovak *et al.* 2014). The GWAS was performed on a GMI computer cluster with algorithms identical to the ones used in the GWAPP Web interface (Seren *et al.* 2012). In particular, this is an accelerated mixed model (EMMAX) (Kang *et al.* 2010) followed by EMMA (Kang *et al.* 2008) for the most significant 200 associations. We took into account SNPs with minor allele counts greater than or equal to 12.

#### Identifying genes with conservation of root angle Between A. thaliana and Solanum species:

The GWAS results for median root direction index/root angle were filtered to include only SNPs that passed FDR threshold of 10% using the Benjamini–Hochberg–Yekutieli multiple testing procedure (Benjamini and Yekutieli 2001). We listed annotated *A. thaliana* genes (Lamesch *et al.* 2012; TAIR10 2013) that were within 4000 bp (upstream or downstream) of significant SNPs. This list was intersected with putative *S. lycopersicum* orthologs that mapped into intervals that influenced root GSA from ILs significant for avgAbsAng. Putative orthologous genes were selected through a multi-step process utilizing the expressolog database (Patel 2013; Patel *et al.* 2012). *S. lycopersicum* genes having an expressolog homology score of at least 50 and an expression correlation of at least 0.25 were selected. If these conditions could not be met, the *S. lycopersicum* gene with the highest homology score in the same orthoMCL (Li *et al.* 2003) cluster as the *A. thaliana* gene and having a homology score of at least 50 was used.

#### Growing and phenotyping T-DNA and transgenic Lines for GSA:

Col-0 and randomly drawn (negative control) T-DNA lines in Table S7 were grown and phenotyped for GSA. Confirmed homozygous lines were obtained using PCR with the standard primers for the particular T-DNA and transgenic line (Table S8). For each line tested, two plates of seedlings were grown. Each plate had 16 T-DNA or transgenic seeds and 16 Col-0 seeds on opposite sides of the plate with four rows of four seeds each, and were grown in the same growth chamber for seven days, then scanned on an Epson Perfection V700 photo flatbed scanner into 24-bit RGB TIF image files at 600 dpi.

#### Fixed-effect linear models for root angle analysis of T-DNA and transgenic lines:

Scanned images were loaded into ImageJ (Rasband 1997-2014) supplemented with custom macros as described for the tomato root imaging. Angle traits were computed and used to fit the fixed-effect linear model “angle ∼1+genotype*plate” on an individual basis for the seedlings from each pair of plates containing a single T-DNA or transgenic line and Col-0 controls, using the R functions lm() and anova().

## Data availability statement

RNA-seq raw read data for the ILs and parents are available under GEO accession GSE87162. Supplemental material available at Figshare: https://doi.org/10.6084/m9.figshare.7237562.

## Results

### Long-term and short-term regulation of root growth angle differs Between M82 and S. pennellii

*S. lycopersicum* cv. M82 differs dramatically from *S. pennellii* in mean root tip angle (Figure 1A) (Ron *et al.* 2013). To determine whether this difference extends to the net root angle, we used a more sensitive and quantitative descriptor – average absolute angle (avgAbsAng). We defined avgAbsAng as the net angle from the root-hypocotyl junction to the final root tip (Figure 1B). This is a different trait than root slanting or skewing. Slanting or skewing in Arabidopsis is characterized by a right-handedness of growth observed on a vertical plate but which is enhanced on a tilted agar plate. In tomato, there is no similar angle bias as neither M82 nor *S. pennellii* displayed a significant preference for right-handed or left-handed growth (Figure S1). The two parents differed significantly (p_FDR_ = 6e-45) in avgAbsAng with *S. pennellii* always having an increased angle relative to *S. lycopersicum* (Figure 1A, 1C).

To explore whether the short-term dynamics of a root’s response to gravity (gravitropism) differs between the accessions of these two Solanum species, root tip angle was measured every 3.75 min at 34 time points over the course of two hours following a 90° rotation (Figure 1D). 3.75 min was determined to be an appropriate sampling frequency such that enough growth occurred during the time interval to exceed one pixel, the minimum measurable change. The standard coordinate system for rotation experiments is used, where 0° is to the right (direction to which seedlings were rotated) and positive angle is clockwise. In response to the 90° rotation, M82 rapidly changed over the 124 min from an average initial angle of -4° to +32° (90-32 = 58° from vertical), a total change of 36° (Figure 1E, Supplemental Movies 1 and 2). In contrast, the mean angle of *S. pennellii* changed much more slowly, from its initial average angle of +10° to +22° (90-22 = 68° from vertical), a change of 12°, two hours after rotation. The initial root angles are not at 0° because the plates were rotated 90° independent of their root’s established growth angles. Thus, if one considers a horizontal line in the changed plate to be 0°, some roots may rotate “through” the gravity vector to a positive angle (below horizontal after rotation) and others will rotate “away” from the gravity vector to a negative angle (above horizontal after rotation). The response curve of tip angle over time has the same shape for roots rotating “away” and “through”, and therefore “away” and “through” samples were combined to calculate the mean rate of change of the root tip angle (swing rate) (Figure 1F). Swing rate measures the dynamics of gravitropism. These dynamics differ substantially between the accessions of these two species. Approximately 30 min (Timepoint 7) after the gravity stimulus, M82 showed a maximum swing rate of 11° min^-1^, while *S. pennellii* showed a peak of 4° min^-1^ at 10 min, followed by a dampened and noisy swing rate, when compared to M82 (Figure 1G, Table S2). ANOVA analysis showed that the swing rate of *S. pennellii* differed significantly (p_FDR_ ≤ 0.01) from M82 at 11 time points (Table S3). To determine if root growth rate over this time period could account for these differences in swing rate over time, we also calculated root growth rates for M82 and *S. pennellii* (Figure S2). These rates are very similar and are not sufficient to explain the difference in the gravitropic response between the accessions of these two species. This is similar to what has been reported in *Arabidopsis thaliana* (Brooks *et al.* 2010).

### Identification of genetic loci regulating the S. pennellii long-term root growth angle trajectory

To explore whether the short-term *vs.* long-term regulation of root angle is determined by distinct genetic mechanisms, we proceeded to map the underlying loci using the introgression lines derived from these two parents (Eshed and Zamir 1995). Out of the 76 ILs, 23 were significantly more angled than M82 (p_FDR_ ≤ 0.01) for avgAbsAng (Table S1) which highlights the multigenic control of this trait. Significant difference of an IL relative to its recurring parent is by definition a QTL. In a previous study, 15 ILs had a significant increase in mean root tip angle (Ron *et al.* 2013) and 11 of these 15 were among the 23 ILs identified as having an avgAbsAng significantly different from M82 (Figure S3). Of the remaining four ILs, 6-1, 6-2-2, 8-2, and 9-1, one (6-2-2) was not grown or measured in this experiment due to lack of seed. We re-measured the archived images of these four ILs for the avgAbsAng trait, and all four were significantly different than M82 (Table S1). Two of these ILs had low but non-significant avgAbsAng q-values in the current experiment (0.175 for 6-1; 0.060 for 8-2) and the last IL, 9-1, had completely non-significant avgAbsAng. This extensive overlap of these two datasets is expected since avgAbsAng and mean root tip angle measure similar traits. We thus included the four ILs that were exclusive to the earlier experiment, to comprise a comprehensive set of ILs regulating root angle (Table S1).

### Identification of genetic loci regulating root gravitropism in the short-term

**Figure 2:**
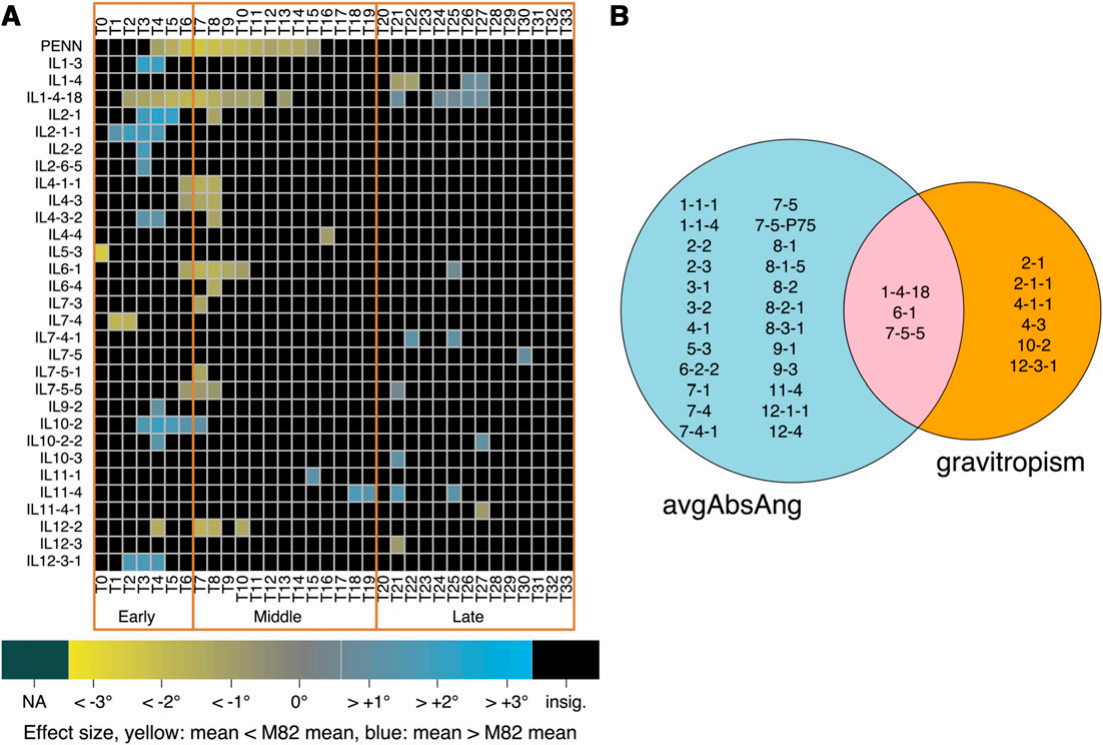
The genetic architecture of root angle is largely distinct from that of root gravitropism. (A) ILs showing significant differences in swing rate over time. Time points are separated by 3.75 min. Early response = T0-T6, middle response = T7-T19; late response = T20-T33. Color indicates magnitude of effect (difference of means of swing rates of ILs *vs.* M82), and only ILs with p_FDR_ ≤ 0.05 (ANOVA) are colored. (B) Overlap in QTL for root angle and QTL for gravitropism at three or more consecutive time points (p_FDR_ ≤ 0.05, ANOVA) relative to M82.

Because these two accessions differ in root angle and gravitropism, we proceeded to map the underlying loci. The same automated pipeline and ANOVA analysis approach described above was used to phenotype root tip angle and swing rate over a two-hour time period as described above, in the same IL population as that for identification of loci regulating root angle. ANOVA statistical models showed that 30 ILs that were significantly different than the M82 recurrent parent in swing rate at many time points (Figure 2A, Table S3). Nine ILs had a significantly different swing rate compared with M82 at three or more consecutive time points, with some ILs responding to the gravity stimulus more slowly than M82 (IL1-4-18, IL4-1-1, IL4-3, IL6-1 and IL7-5-5) and other ILs responding faster than M82 (IL1-4-18, IL2-1, IL2-1-1, IL10-2 and IL12-3-1). Notably, IL1-4-18 was slower than M82 from T2 through T11, and faster than M82 from T24 to T27. Those responding faster than M82 did so either early or late in the time course, while those responding slower than M82 typically did so in the middle of the time course. This shows that distinct genetic loci regulate the root’s rate of change in growth angle at different times over the observed period.

### Common and distinct genetic loci determine short-term and long-term regulation of root angle

We hypothesized that if root angle and gravitropism were controlled by polymorphisms in genes within the same pathways, that the significant ILs identified would overlap between these two traits. Alternatively, if the polymorphisms were in genes affecting different pathways, the ILs would be specific to one trait (*i.e.*, gravitropism) and not affect the other trait (*i.e.*, avgAbsAng). Pearson correlation of avgAbsAng with swing rate at each of the 34 time points showed that R^2^ is never more than 0.2 and is only larger than 0.1 at 2 time points (T21 and T22) (Figure S4). Comparison of significant ILs identified for gravitropism (limited to those with a swing rate significantly different from M82 at three or more consecutive time points) and for root angle (avgAbsAng significantly different from M82) The vast majority of the significant ILs are specific to either root growth angle or gravitropism. Twenty-four ILs are specific to root growth angle, six ILs are specific to gravitropism, and only three ILs (1-4-18, 6-1 and 7-5-5) showed an effect on both root growth angle and gravitropism (Figure 2B). Therefore, root angle and gravitropism are largely regulated by different loci suggesting that most of the polymorphisms are trait-specific. We hypothesize that the two ILs which influence both traits may contain loci that influence common molecular processes that are central to a plant’s response to gravity.

### Global transcriptional changes underlying root angle and gravitropism

A common approach to identify causal genes underlying QTL is to conduct an Expression Quantitative Trait Locus (eQTL) analysis. To further refine this approach, we conducted a developmentally focused eQTL analysis by measuring gene expression solely within 1 cm of the root tip under the hypothesis that signaling that regulates these traits operates within the root tip. Using RNA-seq, we measured variation in gene expression within 1 cm of the root tip for the parents and all the ILs across independently replicated samples. To align the resulting sequences, we created custom chimeric reference genomes for each IL, predominantly comprised of *S. lycopersicum* sequence except for the IL’s introgression region, which was comprised of *S. pennellii* sequence. This approach has previously been successfully utilized to characterize biological processes underlying leaf number, complexity and hypocotyl length (Ranjan *et al.* 2016; Koenig *et al.* 2013).

**Figure 3:**
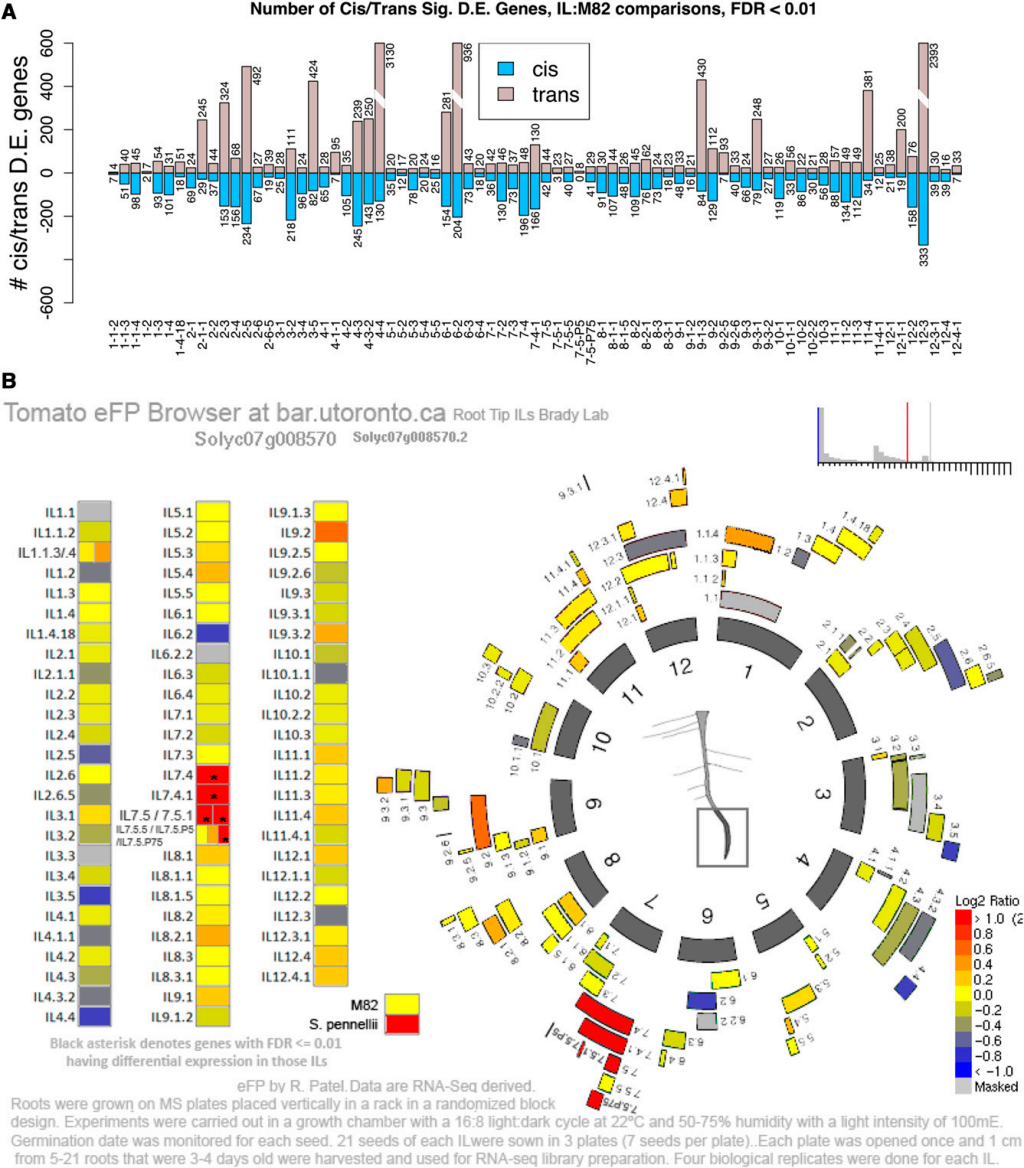
Differentially expressed genes associated with *cis*- and *trans*-eQTL in the root tips of ILs. (A) Number of differentially expressed genes (y-axis) between each IL (x-axis) and M82 at p_FDR_ ≤ 0.01 (negative binomial distribution tests with edgeR). Blue = *cis*-eQTL; Gray = *trans*-eQTL. (B) Tomato eFP browser with chromosome positions of introgressions in ILs. Expression of Solyc07g008570 (*SlPAP27*) is indicated in each IL. Asterisks indicate significant differences from M82 (p_FDR_ ≤ 0.01).

Each identified eQTL represents a position in the genome where a polymorphism causes differential accumulation of a specific transcript. A *cis*-eQTL represents a polymorphism physically located near the gene encoding the transcript being measured. A *trans*-eQTL is located in a portion of the genome with no physical linkage to the gene encoding the measured transcript (Kliebenstein 2009). Differentially expressed genes (DEGs) and thus, eQTL, were identified in *cis* and in *trans* for each IL relative to M82 (Kliebenstein 2009). In the majority of ILs, the number of *cis*-eQTL is more than the number of *trans*-eQTL (Figure 3A). The frequency of *trans*-eQTL was more variable than that of *cis*-eQTL suggesting that ILs with much higher numbers of *trans*-eQTL are hot spots for *trans*-effects (Figure 3A). The resulting differential gene expression in each IL is visualized at the University of Toronto BAR browser (Patel 2015) (Figure 3B).

### Fine mapping of regulatory genes for root angle

**Figure 4:**
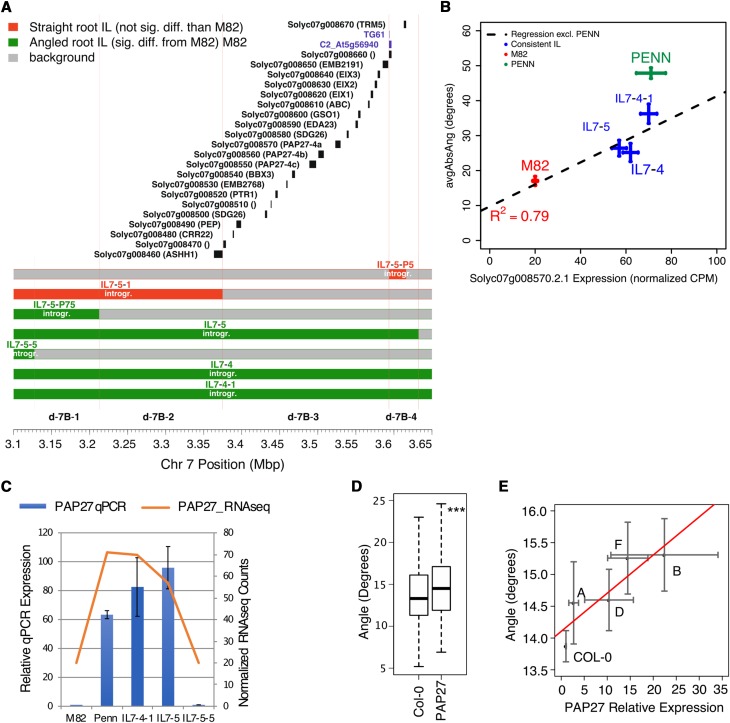
*Solyc07g008570* is localized to a genetic interval associated with regulating root angle and its expression correlates with root growth angle in ILs with introgressions on chromosome 7. (A) Bin d-7B-3 contains 19 genes. Chromosome 7 position (Mbp) is indicated on the x-axis, and each *S. pennelli* introgression within the IL or sub-IL is colored green for a significantly increased avgAbsAng relative to M82 or red if it is not different from M82. Light red lines indicate approximate boundaries of each bin. (B) Expression of *Solyc07g008570* is positively correlated with avgAbsAng using the ILs defining bin d-7B and as determined using RNA-seq (Pearson correlation), error bars are standard error of the mean, n_EXPR_: M82:4, *S. pennellii:*3; IL7-4:4; IL7-4-1:4, 7-5;3; n_ANGLE_: M82:64, *S. pennellii:*78; IL7-4:34; IL7-4-1:38; IL7-5:29. (C) Quantitative real-time PCR confirmation of the increased expression of *Solyc07g008570* in ILs with increased avgAbsAng, n = 3 for each genotype, error bars represent standard deviation. (D) An increase of *AtPAP27* expression is positively correlated with an increased root angle using multiple independent insertion lines (R^2^ = 0.66, *P* ≤ 0.05, ANOVA). Error bars represent standard error of the mean, n(Col-0)=275, n(35S:AtPAP27 ∑A:D)=277. (E) The average absolute angle of four independent insertion lines overexpressing *AtPAP27* is increased relative to Col-0 (**P ≤ 0.01, ANOVA). Error bars represent standard error of the mean, n_Angle_: Col-0:275, 35S:AtPAP27/lineA:11, lineB:20, lineD:83, lineF:100; n_Expr_: Col-0:19, 35S:AtPAP27/lineA:11, lineB:20, lineD:22, lineF:7.

To identify putative regulatory genes controlling the variation in avgAbsAng, we refined the size of candidate genetic intervals by identifying “bins”, areas of overlap covered by multiple ILs (Figure 3B). Among the 23 ILs which have a significantly different root angle than M82, are 49 “bins” which represent independent genetic intervals that regulate avgAbsAng. This allowed us to focus on the bin d-7B interval, defined by overlap of ILs 7-5, 7-4-1 and 7-4, which all possess an increased avgAbsAng and the exclusion of the region defined by IL7-5-5, which did not possess an increased avgAbsAng. To further narrow the bin d-7B interval, we investigated sub-ILs that subdivided this bin, sub-IL7-5-1, sub-IL7-5-P5 and sub-IL7-5-P75 (Figure 4A). The sub-IL7-5-P75 had an average absolute angle that differed significantly from M82, while sub-IL7-5-1 and sub-IL7-5-P5 did not (Figure 4A). This result indicates that sub-bin d-7B-3, which spans an estimated 220kbp region comprising 19 genes, contains a gene for which the *S. pennellii* allele is causative for a higher avgAbsAng. To further reduce the number of candidate genes within these regions, we identified candidate *cis*-eQTL in multiple ILs whose introgression overlaps define a bin where expression correlated with root avgAbsAng. Of these bins with correlated *cis*-eQTL, bin d-7B-3 contains a single *cis*-eQTL out of 19 genes in the interval, *Solyc07g008570*, *PURPLE ACID PHOSPHATASE27-4a* (Figure 4B). Expression of the gene associated with this *cis*-eQTL, *Solyc07g008570/Sopen07g004470 (*hereafter referred to as *SlPAP27)* correlates with root average absolute angle across the ILs used to identify the interval on chromosome 7 (R^2^ =0.79, Figure 4B) and was confirmed with qPCR in these ILs (Figure 4C).

Using gene phylogenetic trees (Figure S5, S6), we identified the nearest *SlPAP27 (designated SlPAP27-4a* in Figure S6*)* homolog in Arabidopsis as *AT5G50400 (AtPAP27)*. To test and validate the sufficiency of *AtPAP27* to regulate root growth angle in plants, we proceeded to test this gene’s influence on Arabidopsis root angle. Since increased expression of this gene was correlated with increased growth angle in *S. pennellii*, we overexpressed *AtPAP27* in the Col-0 accession to determine if this was sufficient to alter root growth angle. The collective average absolute angle of *AtPAP27* summed across four independent overexpression lines of *AtPAP27* differed significantly from Col-0 (*P* ≤ 0.01 including insertion line in the linear model, Figure 4D). Furthermore, avgAbsAng significantly linearly correlates with expression of *AtPAP27* (*P* ≤ 0.01, Figure 4E). Together, these data phenocopy our observations in the tomato introgression lines and their parents where two copies of the *S. pennellii* allele likely confers higher expression of *PAP27* and increased average absolute angle. Root length does not vary linearly with respect to *AtPAP27* expression in these lines and thus cannot explain these differences in root angle (Figure S7). Thus, the *SIPAP27* homolog, *AtPAP27*, is sufficient to regulate avgAbsAng in Arabidopsis and is associated with variation in the same trait in tomato.

### Variation in Root Growth Angle within *Arabidopsis thaliana* and Solanum species

Significant ILs and eQTL mapping linked overexpression of the *S. pennellii* allele of *PAP27* to increased root angle. Using these tomato data, we hypothesized and obtained further support that increased expression of its homolog *AtPAP27* in Arabidopsis is sufficient to regulate root growth angle (Figure 4D,E). We thus hypothesized that there is genetic similarity in natural variation of root growth angle between tomato and Arabidopsis. To test this potential, we analyzed a comparative dataset in Arabidopsis to identify genes that regulate root angle. Large-scale root phenotyping was carried out using the BRAT algorithm (Slovak *et al.* 2014) and two traits relating to gravitropism, “Root Angle” (net angle of the root vector) and “Direction Index” (average pixel-by-pixel deviations from growth relative to the vector of gravity) were measured in 257 *A. thaliana* accessions four days after germination. These 257 accessions showed continuous variation in “Root Angle” and “Direction Index”, thereby allowing quantitative genetic analysis (Table S4, S5). The BRAT algorithm is designed to provide measurements at the throughput found with GWAS. To ensure that “Root Angle” and “Direction Index” provide similar mathematical approximations to avgAbsAng, we measured avgAbsAng in the 5 accessions with the largest and least “Direction Index” values as well as the largest and least “Root Angle” values. These accessions have increased and decreased avgAbsAng respectively (Figure S8). Thus, these three measurements are capturing similar, but slightly different aspects of root angle.

**Figure 5:**
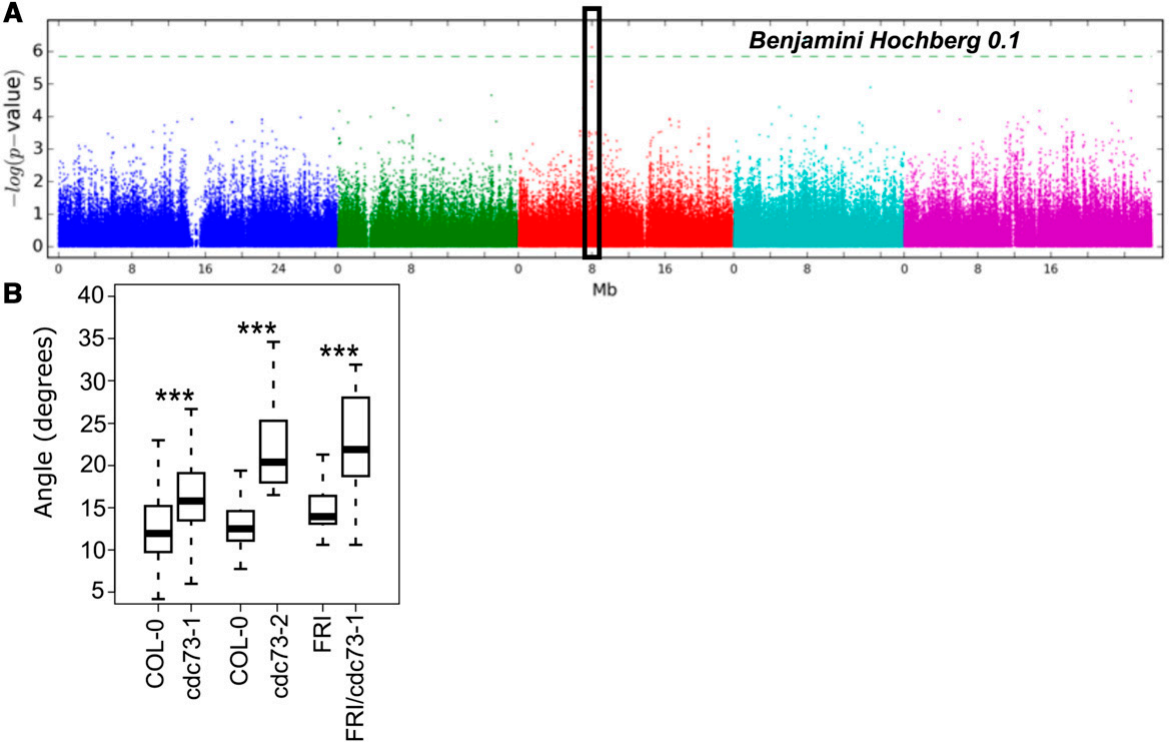
*AtCDC73* regulates root angle in Arabidopsis. (A) GWAS Manhattan Plot for SNPs associated with median direction index. Black box indicates associated genomic region of *CDC73*. (B) Loss of CDC73 function in two alleles (*cdc73-1*, *cdc73-2*) leads to an increased root growth angle relative to Col-0 or the FRI control. *P ≤ 0.05 **P ≤ 0.01 ***P ≤ 0.001 as determined using an ANOVA. n(Col-0)=144, n(*cdc73-1*)=85, n(*cdc73-2*)=26, n(FRI)=22, n(FRI/*cdc73-1*)=32.

A Genome-Wide Association Study (GWAS) was performed to associate single nucleotide polymorphisms (SNPs) with natural phenotypic variation in “Direction Index” and “Root Angle” (Table S4, S5 and Dataset S4). As the GWAS screen was not particularly highly powered with 257 accessions, we chose a stringent but not highly conservative FDR threshold of 0.1. *A. thaliana* genes within 4000 bp (upstream or downstream) of significant (p_FDR_ ≤ 0.1) SNPs (Figure 5A) were intersected with the set of putative *S. lycopersicum* orthologs found within introgression intervals of ILs that were significant (p_FDR_ ≤ 0.05) in avgAbsAng. Using these guidelines, a single gene was associated with “Direction Index” in Arabidopsis, *CELL DIVISION CYCLE 73 (CDC73*, *AT3G22590)* and with avgAbsAng in tomato (*Solyc06g054080 (S. lycopersicum)* and *Sopen06g019240 (S. pennellii))*. This gene was found in bin d-6B, defined by the introgressions contained within ILs 6-1, 6-2 and 6-2-2, all which were significantly different than M82 for avgAbsAng (Figure 2). The significant SNP (*A. thaliana* chromosome 3; position 8008000) for *CDC73* was located 2421 bp downstream of its gene model (Figure S9) and in the upstream region of the *LTPG5* gene. An additional SNP upstream of *CDC73*, that was not included on the original 250K SNP chip used in the GWAS, is common within the haplotype associated with the highest direction index (Figure S9). Using our prior logic that data in tomato may inform our understanding of genes that regulate root angle in Arabidopsis, we carried out experiments to test this the influence of *AtCDC73* on root angle.

Given that avgAbsAng captures the extreme variation observed for “Direction Index” (Figure S8), we measured avgAbsAng in two different TDNA insertion mutant alleles, *cdc73-1* (*php-1*, SALK 150644) and *cdc73-2* (*php-2*, SALK_008357 (Yu and Michaels 2010) as well as in 17 other mutants randomly chosen as a negative control (Table S7, Figure 5B) (Alonso *et al.* 2003; Rosso *et al.* 2003; Sessions *et al.* 2002). Of all these lines, only the mutations in *CDC73* result in an increased avgAbsAng relative to Col-0 (Table S7). The *cdc73-1* allele of CDC73 is also present in the FRIGIDA background and a similar root angle phenotype was identified relative to the FRI control (Figure 5B). While root length was altered in these mutant alleles, these changes were not correlated with the changes in root angle as – *cdc73-1* had a longer root relative to wild type and *cdc73-2* had a shorter root relative to wild type (Figure S10). Thus, changes in root growth are not sufficient to explain these differences in root angle. In summary, these data demonstrate that CDC73 is involved in regulation of root angle in Arabidopsis. Allelic complementation experiments would be needed to resolve which of the polymorphisms identified within the haplotype block are causal for the large avgAbsAng/dirIndex in the accessions.

## Discussion

### Merging quantitative genetic data to identify genes associated With root growth angle

Characterization of root angle in the tomato introgression line population coupled with eQTL analysis in all 76 of these lines allowed the identification of genes associated with a trait of interest. Our finding that the *S. pennellii* allele of *PAP27* is the only gene out of 19 in a significant genetic interval whose elevated expression correlates with increased root growth angle, as well as evidence that *AtPAP27* overexpression is sufficient to increase root angle in *A. thaliana*, are the backbone of our hypothesis that polymorphic alleles of this gene regulate avgAbsAng in all three species. Further evidence is needed to determine if overexpression of the *S. pennellii* allele is sufficient to regulate root growth angle in *S. lycopersicum*. The expression resource of these 76 lines will enable identification of genes for which their altered root expression is associated with a trait of interest. Coupled with a similar resource profiling expression in leaves within this population, genes influencing development pleiotropically across the segregating population can also be identified and should be of use to breeders (Chitwood *et al.* 2013).

To the best of our knowledge, this is the first time that quantitative genetic assays from multiple species (tomato and Arabidopsis) have been combined *de novo* to identify genes regulating root growth angle. Identification of a *CDC73* homolog as regulating root growth angle in Arabidopsis, started with identification of significant ILs in tomato which was subsequently intersected with a GWAS analysis in Arabidopsis. *AtCDC73* was never previously identified to regulate root growth angle in Arabidopsis via developmental genetic screens, suggesting that diverse quantitative genetic datasets across species may be generally effective at identifying genes that naturally vary and thus represent an additional source of novel loci/genes underlying conserved developmental processes. In the GWAS, a single SNP associated with *AtPAP27* was also identified on Chromosome 5 at position 20525997 at an uncorrected p-value of 0.018 (Dataset S4) which did not pass our FDR threshold. The identification of genes regulating root angle in one species based on studies from another species from which its family diverged approximately 112 million years ago (Ku *et al.* 2000), is quite striking.

### Distinct Genetic Regulation of Long-Term and Short-Term Response to Gravity

Many more loci were identified that contribute to regulation of root growth angle (24 QTL/significant ILs or 49 “bins”) and the gravitropic response (6 ILs). These additional loci contribute to the differences in root angle and gravitropism observed between tomato and *S. pennellii*. Although the effect size of the observed change on average absolute angle in *Arabidopsis thaliana* is small within the overexpression lines, it is consistent with the multi-genic control of this trait. Since a greater number of distinct ILs regulating root angle and gravitropism were identified relative to common loci, the genetic architecture of root angle and gravitropism are largely different.

### Acid Phosphatases and their Potential Ecological Importance in Regulating Root Growth

Acid phosphatases, including purple acid phosphatases, are generally up-regulated during the phosphate stress response (PSR) (Żebrowska *et al.* 2011; Bozzo *et al.* 2006) to produce, transport, or recycle Pi (Duff *et al.* 1994). These enzymes are also secreted to scavenge external phosphate from the rhizosphere (Bozzo *et al.* 2002; Abel 2011; Robinson 2012) or localized within cells to serve as Pi transporters (Zimmermann *et al.* 2004; Liu *et al.* 1998). Phosphate is concentrated in upper soil layers, and is often a limited resource, so plants that are seeking phosphorus will develop a root system to locate it via topsoil foraging (Lynch and Brown 2001). *S. pennellii* is endemic to sandy coastal regions and dry rocky regions of Peru and Chile (Moyle 2008; Peralta and Spooner 2005). One hypothesis is that in this region, phosphorus is in low abundance and concentrated near the surface, and *S. pennellii* has adapted by developing a shallow root system architecture to obtain phosphate and capture adequate water during brief (and rare) precipitation events.

### Chromatin Availability, Transcription and Root Growth Angle

*CDC73* (also know as *PHP*, *PLANT HOMOLOGOUS TO PARAFIBROMIN*) (*A. thaliana AT3G22590*, *S. lycopersicum Solyc06g054080*, *S. pennellii Sopen06g019240)* is a promising new candidate for regulation of root growth angle. *CDC73* is a subunit of the Paf1c complex and is a transcriptional co-factor of RNA Polymerase II (Amrich *et al.* 2012). In animals, *CDC73* coordinates various steps of the transcriptional process from initiation to termination by interacting with and recruiting various proteins to the proper locus at each step (Zhang *et al.* 2009; Nordick *et al.* 2008; Shi *et al.* 1997). In Arabidopsis, *CDC73* coordinates the transcriptional regulation and histone post-translational modification of flowering time genes (Park *et al.* 2010). While *CDC73* is expressed in Arabidopsis roots, its function there has never been described. Rapid transcriptional changes accompany the response of Arabidopsis roots to a gravity stimulus and we propose that *CDC73* may influence chromatin availability dynamics and/or transcriptional regulation of critical genes during the response to a gravity stimulus (Kimbrough *et al.* 2005; Kimbrough *et al.* 2004). While all SNPs are synonymous within the CDC73 coding region between *S. lycopersicum* and *S. pennellii*, extensive changes exist in both the 5′ and 3′UTR and in the intron-exon structure, suggesting altered transcriptional or translational regulation may influence root growth angle.
